# SARS-CoV-2–host cell surface interactions and potential antiviral therapies

**DOI:** 10.1098/rsfs.2020.0081

**Published:** 2021-12-10

**Authors:** Aura-Bianca Butnariu, Alex Look, Marta Grillo, Tanveer A. Tabish, Michael J. McGarvey, Md Zahidul I. Pranjol

**Affiliations:** ^1^ School of Life Sciences, University of Sussex, Falmer, UK; ^2^ Faculty of Engineering, Department of Materials, Royal School of Mines, Imperial College London, London, UK; ^3^ Department of Infectious Disease, Faculty of Medicine, Imperial College London, London, UK

**Keywords:** COVID-19, spike protein, ACE2, antivirals, monoclonal antibody

## Abstract

In this review, we reveal the latest developments at the interface between SARS-CoV-2 and the host cell surface. In particular, we evaluate the current and potential mechanisms of binding, fusion and the conformational changes of the spike (S) protein to host cell surface receptors, especially the human angiotensin-converting enzyme 2 (ACE2) receptor. For instance, upon the initial attachment, the receptor binding domain of the S protein forms primarily hydrogen bonds with the protease domain of ACE2 resulting in conformational changes within the secondary structure. These surface interactions are of paramount importance and have been therapeutically exploited for antiviral design, such as monoclonal antibodies. Additionally, we provide an insight into novel therapies that target viral non-structural proteins, such as viral RNA polymerase. An example of which is remdesivir which has now been approved for use in COVID-19 patients by the US Food and Drug Administration. Establishing further understanding of the molecular details at the cell surface will undoubtably aid the development of more efficacious and selectively targeted therapies to reduce the burden of COVID-19.

## 1. Introduction

Coronavirus disease 2019 (COVID-19) is caused by a novel strain of coronavirus (CoV), termed severe acute respiratory syndrome (SARS)-CoV-2. First identified in December 2019 in Wuhan, China, the infection has since spread globally. The wide and rapid spread of the disease led the World Health Organization to recognize the outbreak as a pandemic on 11 March 2020 [1], which is still ongoing. SARS-CoV-2 has infected more than 118 million people worldwide resulting in 2.6 million deaths.

Coronaviruses are large enveloped, non-segmented, positive-sense RNA viruses. There are four coronaviruses that circulate in humans which are historically known to cause mild respiratory diseases: two α-coronaviruses (NL63 and 229E) and two β-coronaviruses (HKU1 and OC43) [2]. In the past two decades, there have been two cases of crossovers from animals to humans which have resulted in severe disease. In late 2002, a new coronavirus, with origin in horseshoe bats, designated as SARS-CoV-1, caused an epidemic that appears to have started in the Guangdong province of China. SARS-CoV-1 affected 8422 people and caused 916 deaths (mortality rate 11%) before being contained [3]. A decade later, another novel coronavirus, also from bat origins, emerged in the Saudi Arabia causing the Middle East respiratory syndrome (MERS). MERS-CoV affected 2494 people of which 858 died (mortality rate 34%) [4].

The genome of SARS-CoV-2 shares a sequence identity of 80% with SARS-CoV-1. Furthermore, it has been shown that SARS-CoV-2 uses the same receptor to enter host cells—angiotensin-converting enzyme 2 (ACE2)—as SARS-CoV-1 [5]. Entry into the host cell is a crucial and necessary step in the life cycle of the virus. In fact, it is the initial interaction with the host cell that allows the virus to enter, establish an infection and replicate, which can lead to tissue damage and ultimately death in some cases. Due to this similarity, much of the understanding of SARS-CoV-2 molecular interactions, proteins and pathogenesis has been based on research in other coronaviruses, especially SARS-CoV-1. In this review, we explore SARS-CoV-2–host cell surface interactions, the cellular entry mechanisms, potential therapies and allude to why SARS-CoV-2 has developed pandemic potential when compared with other coronaviruses.

## 2. Structural proteins

**Figure 1.**
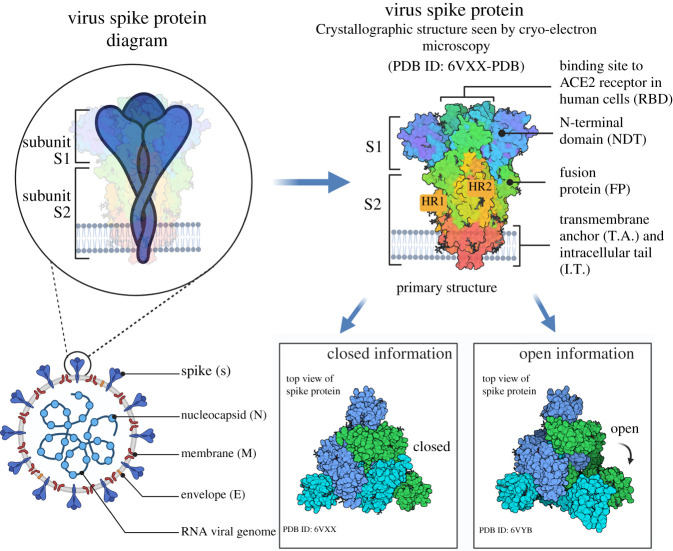
SARS-CoV-2 structure and the structure of the spike protein. The newly discovered SARS-CoV-2 comprises a lipid envelope from the host with four viral structural proteins including the spike (S), envelope (E), membrane (M) and nucleoprotein (N) protein. Encased in the envelope is the positive-sense RNA genome of 29–30 kb in size. SARS-CoV-2 uses the human angiotensin-converting enzyme 2 (ACE2) to bind to host cells and to mediate membrane fusion. The S protein comprises two distinct subunits, S1 subunit and S2 subunit. The S2 subunit is a trimeric helical stalk with two heptad repeat (HR) regions HR1 and HR2. The S2 subunit is capped by the clove-shaped trimeric S1 head. The S1 subunit contains the RBD which binds to the ACE2 receptor. The initial attachment leads to the adoption of an open conformation that is thought to facilitate membrane fusion.

Coronaviruses are named for the large spikes protruding from their spherical envelope giving them a ‘crown’-like shape when viewed under an electron microscope. SARS-CoV-2 consists of a lipid envelope from the host with four viral structural proteins, including the spike (S), envelope (E), membrane (M) and nucleoprotein (N) with around 16 non-structural proteins (nsp1–16) and 8 accessory proteins (figure 1) [6,7]. Within the envelope is a single-stranded, positive-sense RNA genome of 29–30 kb in size that is split up into 11 open reading frames which express 11 genes [7–9]. The S protein is a glycoprotein which is pivotal in initiating binding of the virion to the host cell. The S protein is structurally categorized as a class I viral fusion protein that is heavily *N*-glycosylated with two distinct cleavage sites. The M protein works in concert with E, N and S proteins to form the viral structure and plays a major role in RNA binding [10]. The M protein is a long (222 amino acids) structural transmembrane dimer with an N-terminal ectodomain and a C-terminal endodomain. The M protein is the most abundant viral protein and helps maintain the membrane curvature [11]. The M protein together with the much less abundant E protein (75 amino acids) makes up the virus-like particle and has an important role in morphogenesis and release [6,12]. The E protein is a transmembrane protein, also with an N-terminal ectodomain and a C-terminal endodomain. That can oligomerize to create an ion channel, an important function in the virus–host interaction [13]. Finally, the N protein is part of the nucleocapsid and is highly phosphorylated to increase its RNA-binding affinity. The N protein packages the viral RNA into the ribonucleocapsid by binding to the approximately 140 amino acid long RNA-binding domain [14].

## 3. Mechanism of binding and fusion with host cell surface

SARS-CoV-2 is primarily transmitted via airborne droplets expelled from infected persons. Its initial tropism is towards the pneumocytes of the lungs which express ACE2, the receptor SARS-CoV-2 requires for cellular entry [15]. Particularly type II alveolar cells appear to have the greatest concentration of ACE2 within the respiratory tract [16]. However, ACE2 expression is not restricted to the respiratory tract and is expressed in a plethora of other sites including the small intestine, testis, kidneys, heart, thyroid, adipose tissue, colon, liver, bladder and adrenal glands as well as at lower levels in the blood, spleen, bone marrow, brain, blood vessels and muscle [17–19]. In some cases, a threefold higher ACE2 expression has been found in the pancreatic islets compared to the lung [19], suggesting the potential for the virus's targeting specificity for endocrine cells within the pancreas [20,21]. ACE2 expression levels are higher within males; however, the effect of ageing may only effect females [22]. ACE2 is best known for its role for maintaining the homeostasis of renin–angiotensin system by acting as a negative regulator degrading angiotensin II to angiotensin, although it also acts as a chaperone molecule for amino acid transport and the integrin ligand and, as noted, a receptor for SARS-CoV-2 [22]. Unfortunately, the entry of SARS-CoV-2 into the cells through membrane fusion markedly downregulates ACE2 receptors; with loss of the catalytic effect of these receptors, side effects ensue, such as increased pulmonary inflammation and coagulation [23]. ACE2's catalytic site is exposed to circulating peptides and is regulated by both its rate of expression and cleavage from the cell surface [24]. ACE2 catalytic site contains an N-terminal peptidase domain which acts as a carboxypeptidase. The active sites (S2′–S2 subsites) are highly conserved with the carboxy terminus of peptide ligands binding strongly to conserved residues in the S2′ subsite [25].

Upon SARS-CoV-2 encountering a susceptible host cell, the SARS-CoV-2 S protein facilitates entry into cells and is a key determinant of tissue tropism, virus infectivity, pathogenesis and host range [18,26]. Electron microscopy studies show that the S protein is a clove-shaped trimer with three S1 heads and a trimeric S2 stalk, all of which are essential for attachment, fusion and cellular entry [27,28]. The S1 domain mediates receptor recognition and contains two large subdomains, the N-terminal domain often binding sialic acid and the receptor binding domain (RBD) required for ACE2 binding [29]. The C-terminal S2 subunit is responsible for membrane fusion and contains a highly conserved fusion peptide (FP) and two heptad repeat regions; heptad repeat 1 (HR1) and heptad repeat 2 (HR2). The structural features of the S2 domain of CoVs indicate the use of a type I fusion protein system like the well investigated fusion proteins of influenza and HIV viruses; however, the S proteins of CoVs are much longer and appeared to be more complex [30].

The S protein exists in a closed form within the viral membrane with the RBD capping the top of the S2 core. The process of cellular entry begins with the S protein transitioning from a metastable state into a post-fusion conformation after distinct conformational changes [29]. Exposure of the RBD occurs after one S1 component opens exposing the RBD for interactions with ACE2 (figure 1) [29,31,32].

The S protein binds to the extracellular protease domain (PD) of ACE2 which is distinct from the ACE2 catalytic site forming RBD–PD complex [31,33]. An S1/S2 cleavage site is located at amino acid 667 of the precursor protein and it is completely exposed in the prefusion conformation [34]. Whereas, the S2 cleavage site (S2′) is 130 amino acids from the N-terminus of the S2 subunit and it is completely hidden in the prefusion conformation. The site that is first cleaved is located on a flexible loop of the S1/S2 subunits and is required for binding. The S1 RBD performs hinge-like conformational movements that reduce S1 contacts and un-shields the trimeric S2 core exposing the S1/S2 cleavage site [32]. As a result of this conformational change, the FURIN cleavage site is exposed at the boundary between the S1 and S2 subunits which is composed of 8 amino acids (Arg–Arg–Ala–Arg–Ser–Val–Arg–Ser) between 682 and 689 [31,35]. FURIN cuts after the fourth amino acid between Arg and Ser on the S protein mediating S1/S2 dissociation. On cleavage by FURIN between the S1 and S2 domain, the proportion of trimers in an open conformation increases, which facilitates S protein binding to ACE2 [29,36]. Open RBD binding to ACE2 leads to more open trimer conformations, successive RBD openings and ACE2 binding. These changes lead to a fully open ACE2 bound form, whereby the trimeric S1 ring remains bound to the S2 stalk though limited contacts via the S1 subdomains [29]. The top of the S2 is now fully exposed and ready to mediate membrane fusion.

Even in SARS-CoV-2 S fur/mut which lacks the FURIN cleavage site, S1/S2 cleavage by FURIN was not necessary for S-mediated entry; hence, it is speculated that FURIN-like proteases may also be able to facilitate this cleavage [31]. The proteomics work of Anand *et al.* shows that the proprotein convertase subtilisin/kexin (PCSK) family members have similar proteolytic activity to FURIN, suggesting that PCSK family members may also carry out this cleavage. These eight amino acids (Arg–Arg–Ala–Arg–Ser–Val–Arg–Ser) are highly conserved among SARS-CoV-2 circulating strains, whereas they are not in non-COVID-19 SARS-CoV-1 S proteins, indicating the significance of this cleavage site [37]. Interestingly, this sequence of peptides is exclusively conserved on the extracellular domain of human ENaC-ɑ, which implies that the SARS-CoV-2 may have specifically evolved to mimic a human protease substrate [37]. Akin to SARS-CoV-2 ENaC-ɑ also requires proteolytic activation via cleavage between Arg and Ser residues. As SARS-CoV-2 uses FURIN for its own cleavage, it is conceivable to hypothesize that ENaC-ɑ activation is compromised and low ENaC-ɑ activity on epithelial surfaces may hamper sodium water reabsorption contributing to the COVID-19 pathology [38].

Another central modification at the host cell interface is the presence of glycans, which are present on both ACE2 and S protein [39,40]. The SARS-CoV-2 spike protein has 22 predicted N-linked glycosylation sites and 3 *O*-glycosylation sites [39–41], while ACE2 presents six sequences for N-linked glycosylation at its N-terminal extracellular domain and a few potential O-linked sites [42]. Glycosylation on the S protein and ACE2 receptor indicates a possible role in the binding process [43]. For example, Zhao *et al.* suggested a direct glycan–glycan interaction specifically between the glycan at N546 of the ACE2 and the glycans N74 and N165 on the S protein. Furthermore, the glycans of ACE2 at N90 and N322 interact with the protein moiety of the S protein [44]. Recent biochemical and genetic analyses found that mutations in the glycan at N90 on ACE2 increase the susceptibility to SARS-CoV-2 infection by enhancing the binding of the angiotensin receptor to the RBD of the S protein [45–47].

Following receptor binding and conformational changes, the S2 subunit now plays a key role in mediating viral fusion with the host cell membrane. Membrane fusion occurs when closely apposed lipid bilayers merge, forming a continuous single bilayer which allows the transfer of viral RNA into the host cell [30]. Membrane fusion and organization is highly dependent on the presence of calcium ions [48], as well as being influenced by the concentration of cholesterol within the membrane [10,49]. In the process of the RBD transitioning into the open conformation, molecular interactions from the S1 domain with a segment that precedes the S2 FP region are lost; it is hypothesized that this primes the S protein for helical rearrangements of S2 domain required for viral and host cell membrane fusion [29,36]. This is facilitated by the transmembrane protease serine 2 (TMPSSR2) or cathepsin L/B which cleaves the S2′ site exposing the highly conserved FP [50,51]. The FP is required for viral entry into host cells which alters the membrane organization and dynamics of the host membrane to facilitate membrane fusion [49]. The fusion domain comprises four distinct regions, i.e. FP, HR, transmembrane domain (TMD) and cytoplasmic tail (CT) regions [52]. As yet, the role of the CT region is not well established. The FP is a 20–25 amino acid long peptide and is vital for membrane fusion. Mutations along this peptide block fusion mediated viral infection for several viruses [53–55]. HR 1 and HR 2 interact to form a six-helical bundle bringing the viral and host cell membranes together for fusion [56]. The TMD remains anchored to the viral envelope and it is thought that the FP (embedded in the host membrane) interacts with the TMD (anchored in the viral envelope) to facilitate pore formation [57]. In SARS-CoV-1, it has been demonstrated that the site immediately upstream of the FP (S2′) cleavage site or FP1 increases membrane order. Further the sequence downstream of FP1 (FP2) also has characteristics of an active fusion domain. It is suggested that FP1 and FP2 work cooperatively as a bipartite fusion ‘platform’ within an extended FP [48]. The binding of the membranes results in the formation of pores enabling transfer of viral RNA from the viral envelope to the host cell which then replicates in the host cell cytoplasm leading to newly formed genomic RNA.

SARS-CoV-2 high level of infectivity could be potentially due to more efficient membrane fusion to the host cell than other coronaviruses. Sequence analysis of the S protein domains from SARS-CoV-1 and SARS-CoV-2 indicates high levels of sequence homology in both the S1 and S2 domains [58]. Nevertheless, variations within the S2 domains are observed with various novel glycosylation sites present in SARS-CoV-2. At the interface of the receptor binding (S1) and fusion (S2) domains of SARS-CoV-2, there is an extended structural loop containing basic amino acids. It is suggested that this loop confers fusion activation and entry properties and could be a key component in the evolution of SARS-CoV-2 with this structural loop affecting virus stability and transmission [59]. In addition, mutations within the RBD may limit the effectiveness of antibodies targeting this region; thus, predicting which mutations may arise in the RBD may aid in the development of antibody cocktail therapies and aid in vaccine development [60,61].

In addition to SARS-CoV-2 infecting cells of the aforementioned tissues, SARS-CoV-2 has recently been shown to also infect human CD4+ T-helper cells, of severe COVID-19 patients [62]. It was demonstrated that SARS-CoV-2 S protein directly binds to the CD4 molecule, which in turn mediates the entry of SARS-CoV-2 into T-helper cells in a mechanism that also requires ACE2 and TMPRSS2 [62]. Following SARS-CoV-2 entry into T-helper cells, cell function is impaired and interleukin-10 expression is upregulated which is associated with viral persistence, disease severity and the poor adaptive immune response in some COVID-19 patients [62].

## 4. Molecular details of the interactions

SARS-CoV-2 and SARS-CoV-1 S proteins share 77.46% identity, with the major mutations found in the NTD and RBD [63]. The amino acid sequence alignment of SARS-CoV-2 RBD against SARS-CoV-1 RBD indicates the main changes occurred through convergent evolution (table 1). Despite the striking similarities, when compared with SARS-CoV-1 RBD, SARS-CoV-2 RBD binds the PD of ACE2 with more than 10-fold higher affinity, which might explain the increased virus transmissibility and disease severity in humans [31,64,65]. The binding interface formed by SARS-CoV-2 receptor binding motif (RBM) is larger than that formed by SARS-CoV-1 RBM due to structural differences between the two [66]. The formation of new hydrogen bonds between S19 of ACE2 and A475 of the SARS-CoV-2 RBD, as well as Q24 of ACE2 and N487 of the SARS-CoV-2 RBD results in a more compact conformation [43,66]. The binding affinity is affected by K417, which has been found to increase the binding affinity to ACE2 by 2.2 ± 0.9 kcal mol^−1^ when compared with its corresponding V404 of SARS-CoV-1 [67]. Additionally, the interaction between F486 of the SARS-CoV-2 RBM and the hydrophobic pocket of ACE2 (M82, L79 and Y83) is not formed by the corresponding L472 of SARS-CoV-1; this may explain the enhanced binding activity of SARS-CoV-2 [66]. Regarding transmission, Q493 has been associated with the civet-to-human transmission, as electrostatic repulsion is reduced with a neighbouring hot spot K31 of ACE2 [26,68]. Moreover, the evolutionary mutation of K403R gives rise to an RGD motif within the RBD which may confer the ability of the virus to be recognized by integrins in alveolar epithelial cells and enhance its infectivity [32,69]. Toll-like receptor 4 (TLR4) has been shown to recognize the S protein of SARS-CoV-2 via hydrogen bond interactions involving ASN409, ASN333, SER386, SER352, HIS431 and ASN361 on TLR4 and SER221, ASN280, THR588, THR208, ASN657 and TYR204 on the S protein [70].

**Table 1.** Amino acid sequence alignment of SARS-CoV-2 RBD against SARS-CoV-1 RBD by BLAST (https://blast.ncbi.nlm.nih.gov/Blast.cgi). The differences between SARS-CoV-1 and SARS-CoV-2 are shown in bold. a.a., number of amino acid.

strain	a.a.	differences in amino acid sequence alignment	a.a.
SARS-CoV 2	387	LNDLCF**T**NVYADSFV**IR**GD**E**VRQIAPGQTG**K**IADYNYKLPDDF**T**GCV**I**AWN	437
SARS-CoV	374	LNDLCF**S**NVYADSFV**VK**GD**D**VRQIAPGQTG**V**IADYNYKLPDDF**M**GCV**L**AWN	424
			
SARS-CoV 2	438	**SN**N**L**D**SKVG**GNYNY**L**YR**LF**R**KSN**L**K**PFERDIS**TEIYQAGST**PC**NGVEGF**NC	488
SARS-CoV	425	**TR**N**I**D**ATST**GNYNY**K**YR**YL**R**HGK**L**R**PFERDIS**NVPFSPDGK**PC**TP-PAL**NC	474
			
SARS-CoV 2	489	Y**F**PL**QS**YGF**QP**T**N**G**V**GYQPYRVVVLSFE	516
SARS-CoV	475	Y**W**PL**ND**YGF**YT**T**T**G**I**GYQPYRVVVLSFE	502

**Figure 2.**
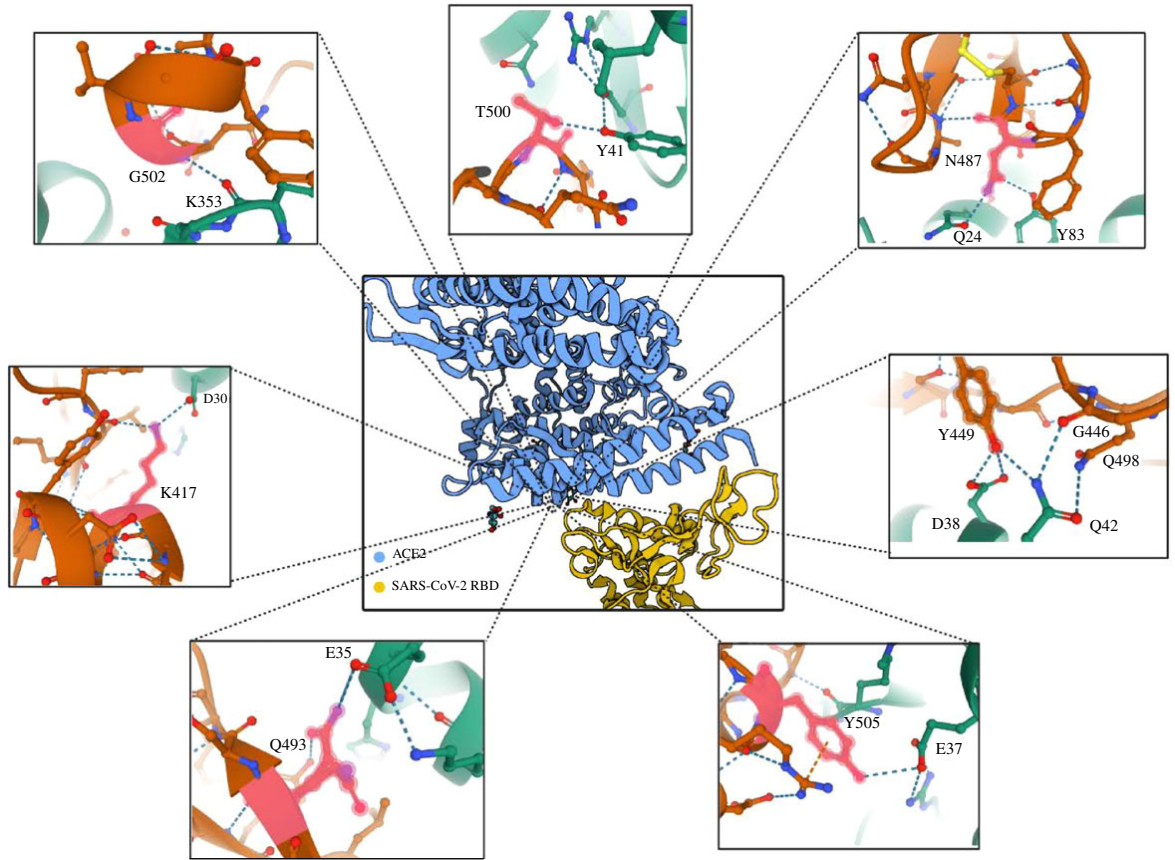
Amino acid interactions at the SARS-CoV-2 RBD–ACE2 interface. The central image depicts ACE2 in blue and SARS-CoV-2 in yellow. Zooming into the interface, the specific hydrogen bonds between SARS-CoV-2 (orange) and ACE2 (green) are shown in blue dotted lines. This figure was created with BioRender.com using images from the RCSB PDB (rcsb.org) of PDB ID 6m0j [42].

Hydrogen bonds are the main interactions that form between SARS-CoV-2 RBD and ACE2 (figure 2) [43,71]. One salt bridge is formed outside RBM between residue K417 of SARS-CoV-2 and residue D30 of ACE2, which is absent in SARS-CoV-1 due to the presence of the corresponding valine [43].

## 5. SARS-CoV-2–ACE2 bound state

The secondary structure of SARS-CoV-2 RBD has revealed a diverse distribution of κ-helices throughout the domain as well as the presence of β-strands, α-helices and 3_10_-helices [71]. Receptor binding is associated with conformational changes at the level of coils and turns, and formation of κ-helices, followed by α-helices, β-strands and 3_10_-helices. The hydrogen bonds of Y495 and the side chains of K443 and Y505 are lost upon binding, leading to the formation of a new κ-helix κ10 and conversion of κ5′ into a coil, which further stabilizes α-helix α5. The side chain of D442 on α5 forms hydrogen bonds and salt bridges with R509, which is rotated and switched to a κ-helix κ12. 3_10_1′ that converts into α1 as a new hydrogen bond is formed between N343 and G339. α1 pulls κ1 forming the N-terminus of the hinge region. Conformational changes occur at the C-terminus of the hinge region as κ14′ converts to β8. A new α-helix α3 is formed as a consequence of Y380 displacement and it forms van der Waals interactions with α2, repositioning the helix one step back. The transition is associated with a small movement of β1 at the medial region of the hinge. The potential of the two pairs of highly conserved cysteines in the hinge region to act as allosteric switches has been evaluated through the assessment of energy, geometrical features of disulfide bonds and quality metrics of cysteine residues [71]. The parallel alignment of cysteines indicated alternating patterns in bond energy, geometrical characteristics and quality between the pairs C336–C361 and C391–C525, which might explain a switch-like mechanism and possible disulfide exchange reactions. These disulfide rearrangements have been previously reported to play a crucial function in triggering membrane fusion process in other viruses such as HIV [72]. Another group showed that the reduction of all disulfide bonds to thiol in both ACE2 and SARS-CoV-2 impairs their ability to bind to each other [73]. However, more structural studies are needed to determine the intricate details of these structural rearrangements.

## 6. SARS-CoV-2–ACE2 unbound state

Recently, cryo-electron microscopy studies have indicated the presence of three pockets within RBD where linoleic acid (LA) molecules can bind [74]. The pockets have a tube-like shape which is lined with phenylalanines, hydrophobic amino acids that form a suitable environment for the hydrophobic tail of LA. A hydrophilic anchor formed by R408 and Q409 interacts with the carboxyl head group of LA, locking the fatty acid inside the pocket. In addition to these molecular features, a gating helix consisting of Y365 and Y369 is localized at the entrance of the hydrophobic pocket, whose role is to open the pocket. In the presence of LA, the gating helix moves away 6 Å allowing the acid to enter the pocket. The hydrophilic anchor moves 10 Å away from the hydrophilic head of LA; once bound to the hydrophobic residues, the RBD trimer compacts triggering the lock down on the headgroup of LA by the anchor. The role of LA is to stabilize the closed conformation of the RBD trimer; therefore, its absence is associated with the unbound open conformation that allows RBD interaction with ACE2 receptor. A surface plasmon resonance assay suggested reduced levels of S binding in the presence of LA. Low levels of LA were found in the serum of people infected with the virus, suggesting LA sequestration by SARS-CoV-2. This essential fatty acid is a precursor of a myriad of molecules that play important roles in cell metabolism and deficiencies were associated with growth-related problems, mental retardation and skin-related disorders in children [75]. More studies suggested that higher plasma levels of LA are associated with a 43% reduced risk of diabetes, decreased plasma levels of serum pro-inflammatory markers, increased levels of anti-inflammatory markers and a reduced risk of cardiovascular disease [75–77]. These facts might offer an explanation for the range of severity issues seen in some patients and their course of the disease [78,79].

## 7. Therapeutic agents

Although specific antiviral treatments are available for coronavirus infections, a lack of specific drugs and vaccines against the new CoV-2 strains has resulted in high mortality rate. One strategy has been to repurpose existing antiviral agents which are known to produce antiviral effect against similar viruses [80]. For instance, ribavirin interferes with nucleic acid metabolism and thus inhibits viral replication, including SARS-CoV-2 *in vitro* [81]. A clinical trial of its efficacy in SARS-CoV-2 patients is currently underway (ClinicalTrials.gov; NCT04356677).

The antimalaria drug chloroquine and its derivative hydroxychloroquine have been shown to have inhibitory activity against a number of viruses in cell culture [82]. Specifically, chloroquine phosphate inhibits terminal phosphorylation of ACE2, while hydroxychloroquine elevates pH in endosomes (involved in virus cell entry) [83,84]. Therefore, chloroquine has the potential to limit and inhibit the *in vitro* spread of SARS-CoV-1 [83]. In recent studies, chloroquine together with hydroxychloroquine inhibited replication of SARS-CoV-2 in Vero cells *in vitro* [85]. Subsequently, chloroquine and hydroxychloroquine were also investigated for their therapeutic efficacy against SARS-CoV-2 in international trials (SOLIDARITY trial) [86]. However, the evidence submitted for hydroxychloroquine versus standard-of-care (SOC) showed that hydroxychloroquine produced no significant reduction in the mortality of hospitalized COVID-19 patients; consequently, this arm of the trial was terminated [87]. In addition, the RECOVEY trial showed that hydroxychloroquine did not reduce the mortality rate of hospitalized COVID-19 patients [88].

Remdesivir had previously been shown to inhibit SARS-CoV-1 and MERS-CoV *in vitro* [89,90] and to inhibit virus levels and lung damage in MERs-CoV-infected non-human primates [91,92]. It was also shown to inhibit SARS-CoV-2 *in vitro* [93] and was subsequently included in clinical trials to evaluate its efficacy in COVID-19 infections. Results show that remdesivir shortened the recovery time of COVID-19 patients who had evidence of lower respiratory tract infections and had been hospitalized (ClinicalTrials.gov; NCT04280705). Due to its high clinical benefit, remdesivir has been recently approved by the US Food and Drug Administration [94].

To successfully enter host cells, SARS-CoV-2 not only has to interact and bind with ACE2 receptors, but also requires priming by TMPRSS2. Studies have revealed that protease inhibitors, such as camostat mesylate, can block the activity of TMPRSS2, preventing viral host cell entry [50]. Consequently, camostat mesylate could be a potential candidate against SARS-CoV-2. Clinical trials are currently ongoing testing the activity of camostat mesylate, combined with SOC treatment, as an inhibitor of TMPRSS2 in patients affected by COVID-19 (ClinicalTrials.gov; NCT04470544).

Other studies suggested that the host cell entry of coronavirus is regulated by receptor-dependent endocytosis. AP2-associated protein kinase 1 (AAK1) is a known regulator of endocytosis, therefore could be considered a target for viral entry inhibition. Studies have revealed that the Janus kinase inhibitor baricitinib is able to inhibit AAK1 and prevent the intracellular assembly of SARS-CoV-2 into target host cells mediated by ACE2 receptor, making it a potential drug candidate against SARS-CoV-2 [95]. A number of clinical trials are currently investigating baricitinib as a possible COVID-19 treatment. One of these clinical trials (ClinicalTrials.gov; NCT04358614) has been completed with encouraging results with a small group of patients showing significantly improved conditions compared to baseline [96].

### 7.1. Monoclonal antibodies

The membrane-anchored spike glycoprotein of SARS-CoV-2 is a key immunogenic antigen which has been shown to be targeted by monoclonal antibodies (mAbs) [97,98]. mAbs may provide a short-term protection from SARS-CoV-2 and help in the fight against the COVID-19. Two specific human mAbs, CA1 and CB6, from COVID-19 patients were isolated that demonstrated *in vitro* potent neutralization against SARS-CoV-2. Specifically, structural studies of these human mAbs revealed that CB6 recognizes the same epitope as ACE2-binding sites in SARS-CoV-2, thus directly competing for its binding [99]. Another neutralizing antibody, CR3022, a SARS-CoV-specific human mAb, was found to potently bind to the RBD domain of SARS-CoV-2 [100]. In this respect, these mAbs may be promising candidates for the therapy of COVID-19.

There are currently several ongoing clinical trials investigating the efficacy of experimental mAbs against SARS-CoV-2 in patients with COVID-19. One of the most promising trials is being conducted by Regeneron Pharmaceuticals (ClinicalTrials.gov; NCT04452318) who are testing a double mAb combination, REGN-COV-2, made of REGN10933 and REGN10987, which is designed to bind at two non-overlapping points on the spike protein of the virus, thus preventing it from entering healthy host cells. Interestingly, the trial is designed to determine if the cocktail of mAbs can also prevent the occurrence of the disease in people exposed to COVID-19 patients, such as healthcare workers. Another trial, conducted by Eli Lilly and Company (ClinicalTrials.gov; NCT04497987), is currently evaluating a mAb isolated from recovered COVID-19 patients, LY-CoV555, to assess its efficacy in preventing SARS-CoV-2 infection in people at high risk and COVID-19 in nursing home residents and staff.

## 8. Future perspectives

As advances have been made in unravelling the molecular biology and pathogenesis of SARS-CoV-2, targeting infection processes, such as attachment to host cell and virus replication, remains of paramount importance in case vaccine design fails. Specific domains within the S protein that can be targeted by antiviral drugs, such as the galectin-like domain and integrin domain, have been recently discovered and are presumed to contribute to virus entry [32,101]. A new type of ganglioside-binding domain on the N-terminus of SARS-CoV-2 protein is thought to interact with sialic acids linked to membrane gangliosides and to have a role in tightening the interaction of S protein with ACE2 [102]. Additionally, the presence of an RGD motif in RBD might confer the ability of the virus to interact with integrins, and it is noteworthy that integrin blockers might prevent virus attachment [69]. More studies are needed to decipher the exact function of these domains and to evaluate if potential inhibitors of these sites affect virus entry. Apart from these interface domains, the LA binding pocket within the RBD can be thought of as a potential allosteric site with great potential for therapeutic targeting, taking into consideration that this approach was considered before for rhinovirus infections [103]. The design of small inhibitors capable of covalent interactions with the pocket and of maintaining the S protein in an irreversibly closed conformation could give rise to a COVID-19 treatment. Additionally, targeting glycans on ACE2 or S protein could potentially lead to the development of therapeutics, such as neutralizing antibodies, that are able to block receptor binding and viral entry of the virus into the host cells.

## 9. Concluding remarks

The surface interactions between SARS-CoV-2 and the host cells are complex and undoubtably there is more to be discovered. ACE2 expression and functional activity is likely to play a key role in the pathology of COVID-19. However, a myriad of host factors including lifestyle, genetics, demographic characteristics and co-morbidities are all likely to influence how effectively the body is able to clear the viral challenge. Deciphering the molecular interactions that occur at the cell surface will enhance our understanding of the entry process, which is likely to lead to an increased number of suitable therapeutic targets which may be pivotal in developing novel therapies to inhibit the virus–host interaction.

## Data accessibility

This article has no additional data.
